# The *MYC* 3′ Wnt-Responsive Element Drives Oncogenic *MYC* Expression in Human Colorectal Cancer Cells

**DOI:** 10.3390/cancers8050052

**Published:** 2016-05-23

**Authors:** Sherri A. Rennoll, Melanie A. Eshelman, Wesley M. Raup-Konsavage, Yuka Imamura Kawasawa, Gregory S. Yochum

**Affiliations:** 1 Department of Biochemistry & Molecular Biology, The Pennsylvania State University College of Medicine, Hershey, PA 17033, USA; sar333@psu.edu (S.A.R.); mhartman3@hmc.psu.edu (M.A.E.); wmk113@psu.edu (W.M.R.-K.); yimamura@hmc.psu.edu (Y.I.K.); 2 Department of Pharmacology, The Pennsylvania State University College of Medicine, Hershey, PA 17033, USA; 3 The Institute for Personalized Medicine, The Pennsylvania State University College of Medicine, Hershey, PA 17033, USA

**Keywords:** *MYC*, Wnt-responsive element, β-catenin, colorectal cancer

## Abstract

Mutations in components of the Wnt/β-catenin signaling pathway drive colorectal cancer (CRC) by deregulating expression of downstream target genes including the *c-MYC* proto-oncogene (*MYC*). The critical regulatory DNA enhancer elements that control oncogenic *MYC* expression in CRC have yet to be fully elucidated. In previous reports, we correlated T-cell factor (TCF) and β-catenin binding to the *MYC* 3′ Wnt responsive DNA element (*MYC* 3′ WRE) with *MYC* expression in HCT116 cells. Here we used CRISPR/Cas9 to determine whether this element is a critical driver of *MYC*. We isolated a clonal population of cells that contained a deletion of a single TCF binding element (TBE) within the *MYC* 3′ WRE. This deletion reduced TCF/β-catenin binding to this regulatory element and decreased *MYC* expression. Using RNA-Seq analysis, we found altered expression of genes that regulate metabolic processes, many of which are known MYC target genes. We found that 3′ WRE-Mut cells displayed a reduced proliferative capacity, diminished clonogenic growth, and a decreased potential to form tumors *in vivo*. These findings indicate that the *MYC* 3′ WRE is a critical driver of oncogenic *MYC* expression and suggest that this element may serve as a therapeutic target for CRC.

## 1. Introduction

The Wnt/β-catenin signaling pathway controls cellular proliferation and differentiation in the intestinal crypt microenvironment [1]. Mutations in components of this pathway are highly prevalent in spontaneously arising colorectal cancers (CRCs) and lead to deregulated expression of genes controlled by the β-catenin transcriptional co-activator [1,2]. β-Catenin is recruited to Wnt responsive DNA regulatory elements (WREs) through interaction with members of the T-cell factor/Lymphoid enhancer factor (TCF/Lef; hereafter TCF) family of sequence specific transcription factors [3]. The TCF7L2 family member is highly expressed in intestinal cells [4,5]. An understanding of colorectal carcinogenesis requires the identification of Wnt/β-catenin target genes and the WREs that control their expression.

The *c-MYC* proto-oncogene (*MYC*) encodes a transcription factor that stimulates cellular proliferation and growth by controlling expression of genes whose products regulate metabolism, ribosome biogenesis, and cell cycle progression [6,7]. He *et al.* identified *MYC* as a direct Wnt/β-catenin target gene in a human CRC cell line and mapped a 5′ WRE within the *MYC* proximal promoter region [8]. Two consensus TCF-binding elements (TBEs) were shown to contribute to 5′ WRE activity [8]. Following this seminal report, a model was proposed, whereby deregulated *MYC* gene expression by oncogenic Wnt/β-catenin signaling was an underlying cause of CRC. However, it was unknown at that time whether the 5′ WRE was the only WRE that controlled *MYC* in CRC, or whether there were additional, yet unidentified *MYC* WREs.

By sequencing DNA isolated in β-catenin chromatin immunoprecipitation (ChIP) assays conducted in the HCT116 human CRC cell line, we previously identified a robust β-catenin binding site that mapped 1.4-kb downstream from the *MYC* transcription stop site [9,10]. This binding site demarcated a *MYC* 3′ WRE that, like the 5′ WRE, also contained two consensus TBEs [11]. Mutation of either TBE reduced *MYC* 3′ WRE enhancer activity when assayed using standard luciferase reporter plasmids [11]. Furthermore, we demonstrated that TCF7L2/β-catenin complexes bound to the *MYC* 3′ WRE coordinated a chromatin loop with the *MYC* 5′ proximal promoter [12]. This chromatin conformation required TCF7L2/β-catenin transcription complexes and correlated with *MYC* expression in these cells.

In this report, we tested whether the *MYC* 3′ WRE controls oncogenic *MYC* expression in the HCT116 human CRC cell line. Using CRISPR/Cas9 gene editing, we isolated a clonal population of cells containing a mutation that deleted the first of two TBEs within the *MYC* 3′ WRE. We found that mutating TBE1 reduced TCF7L2 and β-catenin binding to the 3′ WRE and compromised *MYC* expression. RNA-Seq analysis of control and 3′ WRE-Mut cells, found genes controlling metabolic processes were differentially expressed in knockout cells, most of which are known MYC targets. Finally, when compared to control cells, 3′ WRE-Mut cells displayed reduced cellular proliferation, clonogenic growth, and growth as tumors in a mouse xenograft model. Thus, our findings indicate that the *MYC* 3′ WRE is required for oncogenic *MYC* expression in a human CRC cell line.

## 2. Results

### 2.1. Generation of a 3′ WRE-Mut Cell Line

**Figure 1:**
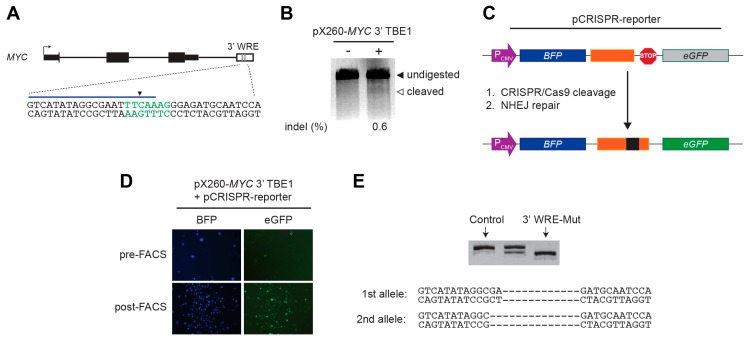
Generation of the 3′ WRE-Mut cell line. (**A**) Diagram of the *MYC* gene with the 3′ WRE depicted by an open rectangle and two vertical grey lines corresponding to the TBE motifs. A strong potential CRISPR/Cas9 cleavage site that overlapped with TBE1 was identified using the CRISPR Design tool [15]. In the expanded view, the TBE1 motif is in green text, the region targeted by the CRISPR guide RNA is depicted by a blue line and the predicted cleavage site indicated by an inverted triangle; (**B**) synergel/agarose gel showing cleavage products obtained from SURVEYOR assays conducted on HEK293T cells transfected with the pX260-*MYC* 3′ TBE1 plasmid; (**C**) diagram of the pCRISPR-reporter plasmid with the *MYC* 3′ WRE target region depicted by an orange rectangle. When the reporter is cleaved by *MYC* 3′ WRE-specific CRISPR/Cas9, repair of the plasmid by NHEJ results in eGFP expression; (**D**) images of HCT116 cells transfected with pCRISPR-reporter and pX260-*MYC* 3′ TBE1 before and after FACS sorting; (**E**) polyacrylamide gel of PCR products generated from HCT116 single-cell clones subjected to CRISPR/Cas9 gene editing. Below, sequence of the targeted alleles in 3′ WRE-Mut cells displaying deletions in TBE1.

To determine whether the *MYC* 3′ WRE is required for *MYC* expression, we first sought to target mutations within this element using CRISPR/Cas9 gene-editing methodology. We identified a potential cleavage site that overlapped the first of two TBEs and engineered the pX260 CRISPR/Cas9 plasmid to express a 3′ TBE1-specific guide RNA (Figure 1A). As a control, we first tested the ability of CRISPR/Cas9 complexes expressed from this plasmid to cleave the *MYC* 3′ WRE in HEK293T cells, which is a cell line that can be efficiently transfected. When this plasmid was expressed in HEK293T cells, CRISPR/Cas9 cleaved the *MYC* 3′ WRE; however, the targeting frequency was low (Figure 1B).

To increase the likelihood of isolating a clonal cell line, we constructed a surrogate reporter plasmid designed to enrich for cells expressing active CRISPR/Cas9 complexes that targeted TBE1 [13,14]. When this plasmid is cleaved by CRISPR/Cas9, and repaired by error-prone non-homologous end joining (NHEJ), transfected cells will express green fluorescent protein (GFP) (Figure 1C). Indeed, after sorting HCT116 cells transfected with the *MYC* TBE1-specfic CRISPR/Cas9 plasmid and the reporter, we were able to enrich for GFP^+^ cells (Figure 1D). We seeded these cells as single clones in each well of a 96-well plate, expanded them, and then surveyed isolated genomic DNA by PCR to identify potential clones containing a mutation within TBE1. We identified a clone that contained a 12-bp deletion on one allele, and a 14-bp deletion on the second allele, each of which deleted TBE1 (Figure 1E). We refer to this clonal population of cells as 3′ WRE-Mut, and the parental population of HCT116 cells from which they were derived as control cells.

### 2.2. Deleting TBE1 Reduced TCF7L2/β-catenin Binding to the MYC 3′ WRE and MYC Expression

**Figure 2:**
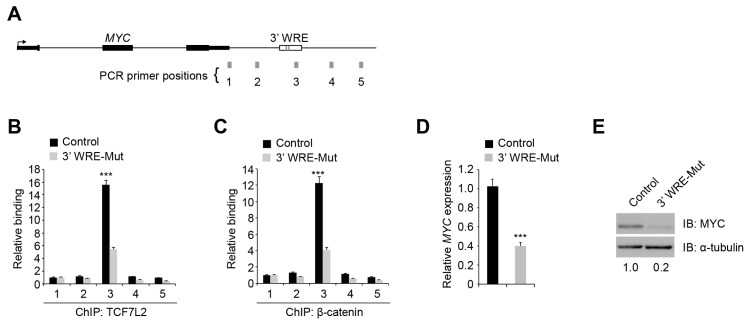
Deleting TBE1 reduces TCF7L2/β-catenin binding to the *MYC* 3′ WRE and *MYC* expression. (**A**) Diagram of the *MYC* genomic locus with the positions of PCR amplicons indicated by grey rectangles and labeled 1-5; (**B**) qPCR analysis of DNA fragments precipitated with anti-TCF7L2 antibodies in ChIP assays conducted in control and 3′ WRE-Mut cells. Shown are signals obtained from PCR amplicons listed on the x-axis. The data is normalized to levels detected with amplicon 1; (**C**) as in (**B**) except anti-β-catenin antibodies were used in the ChIP assays; (**D**) qRT-PCR analysis of *MYC* transcript levels detected in control and 3′ WRE-Mut cells. The data is normalized to *GAPDH* levels; (**E**) Western blot analysis of MYC protein levels in control and 3′ WRE-Mut cells. The blots were re-probed with anti-α-tubulin antibodies to control for loading. Shown below are relative levels of MYC, which were quantified using densitometry. Error bars are ±SEM (*** *p* < 0.001).

We used chromatin immunoprecipitation (ChIP) assays to assess TCF7L2 and β-catenin binding to the *MYC* 3′ WRE in control and 3′ WRE-Mut cells. After precipitating cross-linked and sheared chromatin, the DNA was purified and subjected to quantitative real-time PCR (qPCR) analysis using primer pairs that tiled a 3.5-kb region encompassing the *MYC* 3′ WRE (Figure 2A). In comparison to control cells, 3′ WRE-Mut cells displayed reduced TCF7L2 and β-catenin binding to the *MYC* 3′ WRE (Figure 2B,C). The residual levels of occupancy are likely due to TBE2, which remains intact in 3′ WRE-Mut cells. Using qRT-PCR analysis, we found that deletion of TBE1 resulted in a 2.5-fold decrease in *MYC* transcript levels (Figure 2D). Western blot analysis indicated that MYC protein levels were reduced 5-fold in 3′ WRE-Mut cells (Figure 2E). We isolated a second clonal population of cells harboring similar mutations that deleted TBE1, and found that in comparison to control, these cells also displayed reduced TCF7L2/β-catenin binding to the *MYC* 3′ WRE and reduced *MYC* expression (Figure S1). These findings indicate that the effects on *MYC* transcription are not confined to a single clonal cell population and that TCF7L2/β-catenin binding to TBE1 drives *MYC* expression in HCT116 cells.

### 2.3. The MYC 5′3′ Chromatin Loop Is Maintained in 3′ WRE-Mut Cells

**Figure 3:**
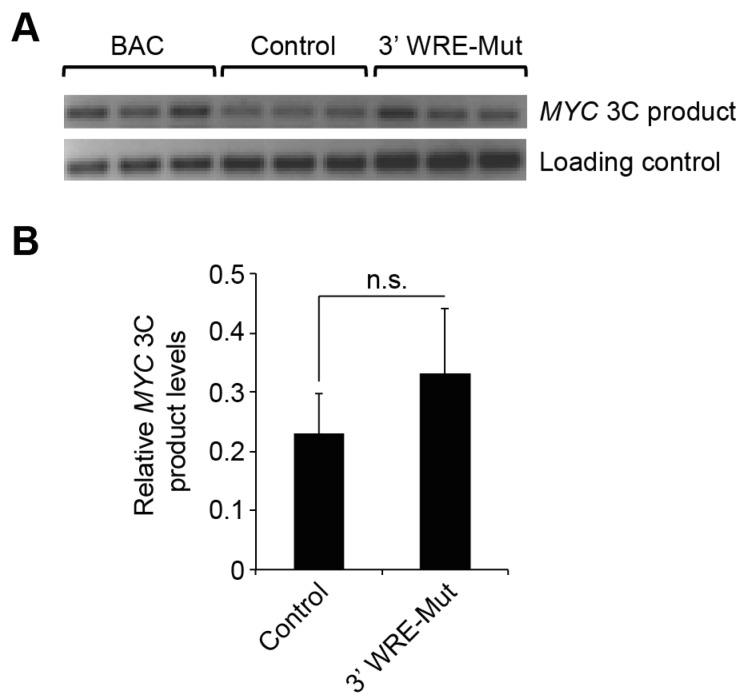
The *MYC* 5′3′ chromatin loop is maintained in 3′ WRE-Mut cells. (**A**) PCR products generated from chromosome conformation capture (3C) analysis conducted in control or 3′ WRE-Mut cells. A bacterial artificial chromosome (BAC) harboring the *MYC* locus was used as a control for 3C. An internal region of *MYC* that is retained in the looped conformation served as a loading control; (**B**) quantification of 3C products. Band intensities were measured from 3 biological replicates, each with 3 technical replicates. n.s., not significant. Error bars are ±SEM.

We have previously shown that TCF7L2/β-catenin complexes bind the *MYC* 3′ WRE and coordinate a chromatin loop with the *MYC* 5′ proximal promoter to drive *MYC* expression in HCT116 cells [12]. We conducted chromosome conformation capture (3C) assays [16] to determine whether formation of the *MYC* 5′3′ chromatin loop is compromised in 3′ WRE-Mut cells. Using a bacterial artificial chromosome (BAC) harboring the *MYC* genomic locus as the template in control 3C assays, we obtained a PCR product of the expected size. Sequencing this product confirmed that it was produced through *MYC* 5′ and 3′ interactions. We then conducted 3C assays in control and 3′ WRE-Mut cells and also assayed an internal region of *MYC* that is retained in the looped conformation as a loading control. Although it appeared that the frequency of *MYC* 5′ and 3′ interaction increased in 3′ WRE-Mut cells relative to control HCT116 cells, this difference did not reach statistical significance indicating that deleting TBE1 did not influence this chromatin conformation at the *MYC* locus in these cell populations (Figure 3A,B).

### 2.4. Transcriptome Analysis of 3′ WRE-Mut Cells

MYC is a transcription factor that regulates expression of thousands of genes involved in cellular proliferation and growth [6,7]. Before analyzing the transcriptome of control and 3′ WRE-Mut cells, we surveyed the genome of 3′ WRE-Mut cells for potential CRISPR/Cas9 off-target mutations (see Materials and Methods and Figure S2). This analysis failed to identify the presence of insertions or deletions at the top sixteen predicted off-target sites, indicating that 3′ WRE-Mut cells likely do not contain spurious mutations (Figure S2). We therefore proceeded to conduct RNA-Seq analysis on transcripts isolated from control and 3′ WRE-Mut cells to understand the potential impact of TBE1 deletion on cellular processes.

**Figure 4:**
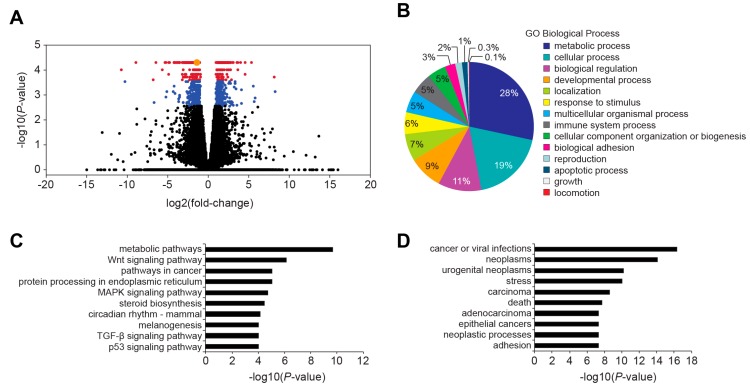
Transcriptome analysis of 3′ WRE-Mut cells. (**A**) Volcano plot showing the log2 (fold-change) for transcripts isolated from 3′ WRE-Mut cells relative to control cells *versus* the −log10 (*p*-value). A total of 949 genes (blue) were differentially expressed at a *q*-value of < 0.05 and 448 genes (red) were differentially expressed at a *q*-value of <0.01. *MYC* is denoted as an orange circle; (**B**) Gene Ontology (GO); (**C**) Kyoto Encyclopedia of Genes and Genomes (KEGG); and (**D**) disease enrichment analyses of genes that were differentially expressed in 3′ WRE-Mut *versus* control cells.

Deleting TBE1 caused a 2-fold or greater change in expression of 949 genes, with 476 targets displaying an increase and 473 targets displaying a decrease in transcript levels (Figure 4A and Table S2). Using a Gene Ontology (GO) enrichment analysis of the 949 differentially expressed genes, we found that over a quarter comprised the “metabolic process” category (Figure 4B). Similarly, a Kyoto Encyclopedia Genes and Genomes (KEGG) analysis of these genes found “metabolic pathways” as the top category (Figure 4C). Interestingly, the Wnt signaling pathway was also enriched in this analysis indicating that MYC is a critical effector of this pathway in these cells (Figure 4C). When classified into disease associations, “cancer” or cancer-related terms accounted for seven of the top 10 categories of differentially expressed genes (Figure 4D). We found by searching the literature that 215 of the 949 genes are either known or predicted targets that are directly regulated by MYC (Table S2). Importantly, roughly half of these genes comprised the metabolic process category in the GO enrichment analysis. Together, these data indicate that deleting TBE1 alters the HCT116 transcriptome and that expression of genes involved in controlling metabolic processes are most affected by the reduced levels of MYC in these cells.

### 2.5. The MYC 3′ WRE Controls the Oncogenic Potential of HCT116 Cells

**Figure 5:**
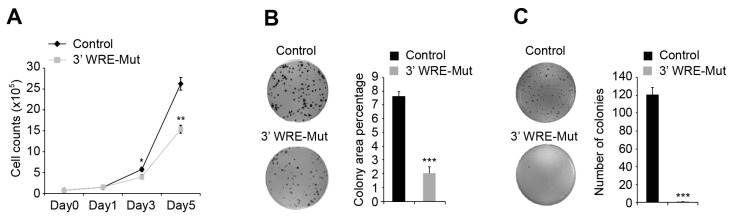
The *MYC* 3′ WRE controls cell proliferation and clonogenic growth. (**A**) Cell proliferation analysis of control and 3′ WRE-Mut cells on days one, three, and five after plating (day 0); (**B**) clonogenic growth of control and 3′ WRE-Mut cells on a plastic substrate. The percentage of area occupied by the colonies was quantified using ImageJ software 10 days after plating; (**C**) anchorage-independent growth of control and 3′ WRE-Mut cells. Colony formation was quantified using ImageJ software 21 days after plating. Error bars are ±SEM (* *p* < 0.05, ** *p* < 0.01, *** *p* < 0.001).

MYC is required for intestinal tumorigenesis in mouse models of CRC [17], and *MYC* levels are elevated in 70% of human CRCs compared to levels seen in un-involved intestinal tissues [18,19]. Because deleting TBE1 reduced *MYC* RNA and protein levels, we tested whether these cells displayed a reduced tumorigenic potential. First, we monitored cellular proliferation and found that in comparison to control cells, 3′ WRE-Mut cells grew more slowly, which was most apparent at day 5 after plating (Figure 5A). Next, we measured the ability of these cells to grow as clones when seeded at low density on a plastic substrate. In line with the proliferation analysis, we noted that plates seeded with 3′ WRE-Mut cells contained a 75% reduction in the number of clones that grew (Figure 5B). A third characteristic of CRC cells is their ability to grow in an anchorage-independent manner. To evaluate this property, we suspended control and 3′ WRE-Mut cells in soft agar, and assessed clonal growth after 21 days in culture. Very few 3′ WRE-Mut clones grew, whereas over 100 were tallied in control cultures, indicating that anchorage-independent growth is diminished in cells harboring TBE1 deletions (Figure 5C).

**Figure 6:**
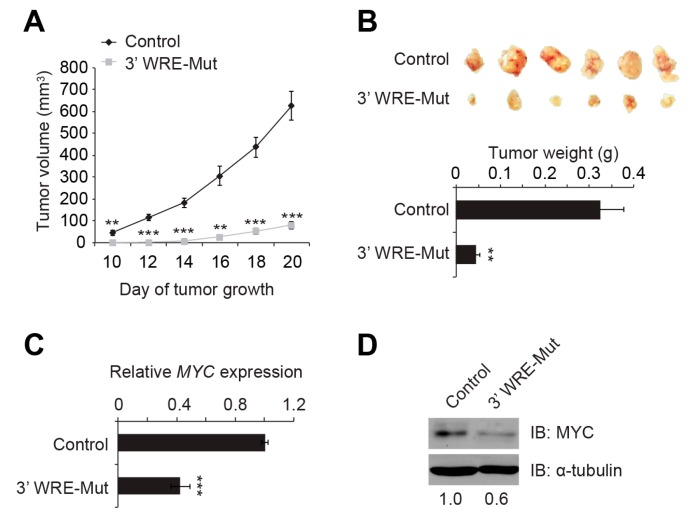
The *MYC* 3′ WRE controls the tumorigenic potential of HCT116 cells *in vivo*. (**A**) Mouse xenograft assays conducted using control and 3′ WRE-Mut cells with tumor volumes indicated on the respective day of measurement; (**B**) images and weights of control and 3′ WRE-Mut tumors that were excised on day 20; (**C**) qRT-PCR analysis of *MYC* transcript levels in tumors explanted on day 20. The data is normalized to *GAPDH* and represented as levels measured relative to that detected in control tumors; (**D**) Western blot analysis of MYC protein levels in control and 3′ WRE-Mut tumors. Shown below are relative levels of MYC that were quantified using ImageJ software and normalized to tubulin levels. Error bars are ±SEM (* *p* < 0.05, ** *p* < 0.01, *** *p* < 0.001).

We next determined whether knocking out *MYC* 3′ TBE1 affected the ability of HCT116 cells to grow as tumors in a xenograft mouse model. We injected control and 3′ WRE-Mut cells subcutaneously into separate flanks of athymic NU/NU mice, and monitored tumor growth with standard calipers beginning on day 10 of the protocol. While 3′ WRE-Mut tumors formed, their onset was delayed, and they contained less volume than tumors in mice that received control cells (Figure 6A). We explanted the tumors at the conclusion of the study and noted that 3′ WRE-Mut tumors were smaller and weighed significantly less than control tumors (Figure 6B). Moreover, 3′ WRE-Mut tumors expressed 2.5-fold less *MYC* transcripts and contained a 40% reduction in MYC protein levels (Figure 6C,D). Together, these results indicate that the *MYC* 3′ WRE promotes CRC cell proliferation and tumor growth by driving *MYC* expression.

## 3. Discussion

Data from The Cancer Genome Atlas (TCGA) project has confirmed that mutations in components of the Wnt/β-catenin signaling pathway occur frequently in CRCs [20]. In most cases, these mutations target the *APC* tumor suppressor leading to inappropriate expression of genes controlled by TCF/β-catenin transcription complexes [2]. Since the discovery of *MYC* as a direct Wnt/β-catenin target gene [8], significant effort has been put forth to identify the WREs that control its expression in human CRC cells. Numerous studies have correlated TCF/β-catenin occupancy at WREs with *MYC* expression in these cells; however, what has been lacking in the field, due to difficulties in genetically engineering somatic cell lines, is whether any of these WREs are required for oncogenic *MYC* expression. In this report, we used CRISPR/Cas9 to delete TBE1 within the *MYC* 3′ WRE in the HCT116 human CRC cell line. We demonstrate that a single TCF binding element embedded within the *MYC* 3′ WRE is a critical regulator of *MYC* expression and that it is required to sustain the full oncogenic potential of these cells.

In our previous β-catenin ChIP-seq screen, nearly half of the high-confidence β-catenin binding regions contained a canonical TCF motif, with most regions containing more than one [10]. Whether each motif contributed to β-catenin recruitment to that region was unknown. Here, we demonstrate that TBEs within a single WRE are not redundant as deletion of TBE1 diminished TCF7L2 and β-catenin occupancy at the *MYC* 3′ WRE by 50% (Figure 2B,C). This indicates that TBE2 is unable to compensate for loss of TBE1. Moreover, sequence analysis of the regions flanking the *MYC* 3′ WRE identified multiple consensus TCF motifs downstream. Our ChIP-qPCR analysis in 3′ WRE-Mut cells failed to detect TCF7L2 or β-catenin binding to these regions (Figure 2B,C). These results clearly demonstrate that multiple TBEs within a single WRE are not functionally redundant and that TCF7L2/β-catenin complexes do not simply shift to occupy other TBEs to compensate for loss of function.

In our 3C analysis, we found that deletion of TBE1 did not affect formation of the *MYC* 5′3′ chromatin loop even though *MYC* expression is significantly reduced in these cells (Figure 3). Thus, this chromatin conformation is not a critical determinant of oncogenic *MYC* expression. However, it is also possible that our 3C analysis lacks the sensitivity to detect small changes in the frequency of the 5′3′ chromatin loop or that the residual levels of TCF7L2/β-catenin binding to the *MYC* 3′ WRE through TBE2 are sufficient to maintain the chromatin architecture. It is also intriguing to speculate that the *MYC* 3′ WRE may affect other aspects of *MYC* expression such as 3′ termination and message processing. Future work will examine this possibility, as well as others, and the 3′ WRE-Mut cells will provide an important resource to tease out the mechanistic details for how this element is operating *in vivo*.

In a previous study, Yao *et al.* reported that deletion of enhancer 7, which is also known as the *MYC* -335 WRE, reduced *MYC* expression 1.5-fold in HCT116 cells [21]. Through RNA-Seq analysis, they found that expression of 105 genes was down regulated in cells lacking this enhancer. We found that deleting TBE1 within the *MYC* 3′ WRE reduced *MYC* expression 2.5-fold and MYC protein levels 5-fold (Figure 2D,E). In our RNA-Seq analysis of control and 3′ WRE-Mut cells, expression of 476 genes was down regulated (Figure 4 and Table S2). Because mutations in TBE1 had a more dramatic effect on *MYC* expression than deletion of enhancer 7, this likely accounts for the discrepancy in the number of targets whose expression levels changed. In addition, it is likely that down-regulated genes are either direct or indirect targets of the MYC transcription factor. Our finding that expression of 215 known or predicted MYC targets was down regulated in 3′ WRE-Mut cells support this conclusion. Together, these results indicate that in comparison to enhancer 7, the *MYC* 3′ WRE is a stronger regulatory element that controls *MYC* expression and the transcriptome in HCT116 cells.

It is also possible that enhancer 7 and the *MYC* 3′ WRE are directly juxtaposed to target genes outside the *MYC* locus through long-range chromatin loops to influence their expression. Indeed, Cai *et al.* found that this is likely the case for enhancer 7 in a circular chromosome conformation capture analysis (4C) analysis conducted in prostate cancer cells [22]. A more thorough investigation of the chromatin architecture of control and 3′ WRE-Mut or enhancer 7-deleted cells using 4C is required to address the potential for these WREs to directly regulate expression of alternative genes in CRC.

Recently, Tak *et al.* found that deleting enhancer 7 compromised HCT116 cell growth [23]. We found that deleting TBE1 reduced HCT116 cell proliferation, clonogenic growth, anchorage-independent growth in soft agar, and tumor growth when these cells were injected into the flanks of immune-compromised animals (Figure 5 and Figure 6). In our RNA-seq analysis of control and 3′ WRE-Mut cells, 215 of the 949 differentially expressed genes are known or predicted MYC target genes (Supplementary Materials Figure S2). These results suggest that *MYC* 3′ TBE1 deletion, and subsequent reduction in MYC protein levels, alters expression of the MYC target gene network. GO and KEGG analyses of differentially expressed targets in control and TBE1-deleted cells found that genes comprising metabolic pathways or processes were the most significantly altered (Figure 4B,C). As MYC has been shown to promote metabolic reprogramming of cancer cells [24], these findings suggest that the reduced oncogenic potential of 3′ WRE-Mut cells is due to an inability to sustain deregulated cellular metabolism.

Studies using mouse models of CRC have shown that MYC is required for intestinal tumorigenesis caused by deregulated Wnt/β-catenin signaling [17]. In the first report that targeted a WRE in the mouse genome, Sur *et al.* found that deleting the *Myc* −335 WRE did not alter intestinal homeostasis [25]. However, *Myc* −335 WRE^−/−^ *Apc^Min/+^* mice contained a reduced number of tumors in both the small intestines and colons when compared to *Apc^Min/+^* mice [25]. In contrast, deleting the *Myc* 3′ WRE in mice altered the crypt microenvironment and increased *Myc* transcript and MYC protein levels in their colons [26]. *Myc* 3′ WRE^−/−^ *Apc^Min/+^* mice contained an elevated number of tumors in the colons in comparison to *Apc^Min/+^* mice [27]. Moreover, deletion of the *Myc* 3′ WRE promoted colitis-associated colorectal cancer in a mouse model of this disease [27]. These findings are seemingly at odds with those reported here, which indicate that the *MYC* 3′ WRE is critical for maintaining the oncogenic potential of a human CRC cell line. There are several possible explanations that may account for these different results. First, while the *MYC* 3′ WRE is highly conserved in mouse overall, TBE1 is not conserved, perhaps indicating that the function of this regulatory element differs between mouse and human [26]. Second, tumors that develop in *Apc^Min/+^* mice and in the colitis-associated cancer model are primarily adenomas, whereas human CRC cell lines are predominantly adenocarcinomas, thereby complicating direct comparisons between the two model systems [28,29]. Third, *Myc* 3′ WRE^−/−^ mice harbor germ-line mutations and deregulated *MYC* expression in cells other than intestinal epithelial cells and these cells may contribute to tumorigenesis. Perhaps the most important difference between these two systems is that in our mouse studies, MYC expression was deregulated prior to the development of tumors whereas in our human cell line studies, these cells were already addicted to high levels of MYC. Regardless, these studies indicate that when considering the function of a regulatory element, interpretation of results obtained from the model system used must be carefully taken into consideration.

With the advancement of gene-editing technologies, the possibility of targeting enhancer elements for therapeutic purposes is within reach [30]. While additional work is required to ensure the specificity of CRISPR/Cas9 complexes in order to reduce potential off target effects [31], our findings indicate that targeting the *MYC* 3′ WRE in human CRCs may be of therapeutic value. While introducing an active DNA nuclease (Cas9) into cells may raise concern, the CRISPR system may be harnessed in other ways to silence an oncogenic element [32,33]. For instance, by introducing multiple guide RNAs that tile the *MYC* 3′ WRE, and a catalytically inactive Cas9 protein fused to the Sin3 interaction domain (SID), one could target the mSin3A/HDAC corepressor complex to this site to repress oncogenic *MYC* expression [34,35]. The key will be to develop specific delivery strategies that target cancer and not the uninvolved colonic epithelium. As colorectal cancer remains a serious worldwide health problem, more creative measures such as these are required to treat this disease.

## 4. Materials and Methods

### 4.1. Cell Culture 

HEK293T (CRL-3216) and HCT116 (CCL-247) were obtained from the American Type Culture Collection (Rockville, MD, USA). Cell lines were cultured in Dulbecco’s Modified Eagle’s Medium (DMEM) supplemented with 10% Fetal Bovine Serum (FBS, Atlantic Biologicals, S11150, Frederick, MD, USA), 50 units/mL penicillin (Corning Mediatech, 30-002-CI, Tewksbury, MA, USA) 2 mM Glutamax (ThermoFisher Scientific, 35050-061, Waltham, MA, USA), and 0.1 mg/mL streptomycin (Corning Mediatech, 30-002-CI) at 37 °C in 5% CO_2_.

### 4.2. Plasmids

The CRISPR Design tool [15] was first used to identify the CRISPR guide RNA (crRNA) sequence within the *MYC* 3′ WRE. DNA oligonucleotides corresponding to this sequence were annealed, phosphorylated using T4 polynucleotide kinase (New England Biolabs, M0201S, Ipswich, MA, USA), and ligated into the BbsI site within the pX260 CRISPR/Cas9 plasmid (Addgene 42229) using instructions on the Genome Engineering Resources website [36]. This plasmid is referred to as pX260-*MYC* 3′ TBE1. The oligonucleotide sequences corresponding to the guide RNA are listed in Table S1.

To design the *MYC* 3′ WRE-specific CRISPR reporter plasmid, we employed a stepwise approach using standard PCR and sub-cloning procedures. The pEGFP-N1 plasmid (Clontech, 6085-1, Mountain View, CA, USA) served as the vector backbone. *BFP* was PCR-amplified from pRSET-BFP (ThermoFisher, V354-20) and the amplified product was inserted as an NheI-EcoRI fragment into pEGFP-N1. Next, the *EGFP* sequence was removed by digesting the vector with BamHI and NotI and replaced with an out-of-frame *EGFP* sequence that was PCR-amplified using an upstream primer that disrupted the reading frame. Finally, a 43-bp oligonucleotide containing the *MYC* 3′ TBE1 CRISPR/Cas9 target sequence was annealed and inserted in between *BFP* and the out-of-frame *EGFP* as an EcoRI-BamHI fragment. This plasmid is referred to as pCRISPR-reporter and the oligonucleotide sequences used for its generation are listed in Table S1.

### 4.3. Mismatch Cleavage Assays

The SURVEYOR mutation detection kit (Transgenomics, 706025, Omaha, NE, USA) was used to evaluate the cleavage frequency of *MYC* 3′ TBE1 CRISPR/Cas9 complexes. Approximately 3 × 10^5^ HEK293T cells were seeded in each well of a six-well dish and transfected with either 500 ng of pX260-*MYC* 3′ TBE1, or 500 ng of pmaxGFP (Lonza, VDF-1012, Köln, Germany) as a control, using Lipofectamine 2000 (ThermoFisher, 11668). The following day, the growth media was supplemented with 0.5 μg/mL puromycin. After two days in selection media, genomic DNA was isolated using the Genomic DNA Purification Kit (ThermoFisher, K0512). A 200 ng quantity of genomic DNA was used as the template in PCR reactions, with primer sequences provided in Table S1, to amplify a 1049-bp region flanking the CRISPR/Cas9 target site within the *MYC* 3′ WRE. The amplicons were purified using the QIAquick PCR purification kit (Qiagen, 28104, Gaithersburg, MD, USA). A total of 400 ng purified PCR product was subjected to rapid denaturation and re-annealing using guidelines provided in the SURVEYOR kit (Transgenomics, 706025). The heteroduplexes were digested with SURVEYOR nuclease and the products were resolved on a 3% synergel/agarose gel (Diversified Biotech, SYN-100, Dedham, MA, USA). ImageJ software was used to measure the band intensities of both undigested PCR products and cleaved fragments. The percentage of insertions/deletions (indels) was estimated using the formula 100 × (1 − [(1 − (*b* + *c*)/(*a* + *b* + *c*)]^1/2^) as described by Ran *et al.* [37].

### 4.4. Generation and Evaluation of HCT116 MYC 3′ WRE-Mut Cells

Approximately 2.5 × 10^5^ HCT116 cells were seeded into a single well of a 12-well dish and the cells were transfected the following day with 375 ng pX260-*MYC* 3′ TBE1, 187.5 ng of pCRISPR-reporter, and 562.5 ng pBlueScript (as carrier DNA) using ViaFect reagent (Promega, E4981, Madison, WI, USA) at a 3:1 ratio of reagent to DNA. Three days later, a BD fluorescent-activated cell sorting (FACS) Aria instrument (BD Biosciences, San Jose, CA, USA) was used to sort single GFP^+^ cells into each well of three 96-well plates. After expansion of single-cell clones, genomic DNA was isolated from each clone using Lyse-and-Go reagent (ThermoFisher Scientific, 78882) and the region flanking the CRISPR/Cas9 target sequence within the *MYC* 3′ WRE was PCR-amplified. The PCR products were analyzed on a non-denaturing polyacrylamide gel containing 8% acrylamide-bisacrylamide (29:1, w/w) and 0.5× Tris-borate EDTA (TBE) to detect differences in band sizes relative to products generated from non-transfected HCT116 parental cells. A single clone was identified that contained a smaller product and sequencing the product identified a 12-bp deletion that removed the entire TBE1 sequence motif on one allele. This cell line was expanded, and the transfection/sorting procedure was repeated to isolate the 3′ WRE-Mut cell line. These cells contained a 14-bp deletion that removed TBE1 on the second allele.

### 4.5. Chromatin Immunoprecipitation (ChIP)

ChIP assays were performed as previously described [10] using approximately 5 × 10^6^ cells per sample. The cross-linked and sheared chromatin was precipitated with 3 μg of anti-TCF4 (TCF7L2) (Millipore, 05-511, Darmstadt, Germany) or anti-β-catenin (BD Transduction, 610154, San Jose, CA, USA) antibodies. The precipitated chromatin was analyzed by quantitative real-time PCR (qPCR) using the primer sets listed in Table S1 and a MyIQ real-time PCR machine (Bio-rad, Hercules, CA, USA). A standard curve was generated using serial dilutions of sonicated and purified input DNA to ensure that ChIP signals were within the linear range of detection. The data is presented as relative levels that were obtained using the standard curves.

### 4.6. Real-Time Reverse Transcription PCR (qRT-PCR)

For experiments measuring *MYC* transcript levels in control and 3′ WRE-Mut cells, 7.5 × 10^5^ cells were plated in a 10 cm dish in a 7 mL volume of DMEM. Total RNA was isolated 5 days later using TRIzol reagent (ThermoFisher, 15596), and cDNAs were synthesized from 500 ng of RNA using the iScript cDNA synthesis kit (Bio-Rad, 1708891) according to the manufacturer’s instructions. To evaluate *MYC* expression in tumors explanted from xenografted mice, a slice of the tumor was homogenized in TRIzol reagent and RNAs were isolated from these samples using instructions provided by the manufacturer. To assess *MYC* transcript levels, cDNA samples were diluted 1:100, of which 3 μL were analyzed in 15 μL qPCR reactions containing 2× Sensimix (Bioline, BIO-96020,Taunton, MA, USA), and 10 pmol each of forward and reverse primers (Table S1). The reactions were cycled using parameters previously described [10]. *GAPDH* levels were measured as an internal reference and the data is presented as relative expression (2^−ΔΔCt^).

### 4.7. Western Blot

Five days after plating 7.5 × 10^5^ control and 3′ WRE-Mut cells in a 10 cm dish with 7 mL DMEM, the cells were collected and the pellet was re-suspended in 400 μL of RIPA buffer (50 mM Tris HCl, pH 8.0; 150 mM NaCl; 1% NP-40; 0.5% sodium deoxycholate; 0.1% SDS) containing freshly added protease inhibitors (1 mM PMSF, 10 μL/mL aprotinin, 10 μg/mL leupeptin). The cells were swelled on ice and briefly sonicated using a Misonix XL-2000 liquid processor (2 × 5 s, output wattage 4, 30 s rest time between pulses). After clarifying the lysates by centrifugation at 16,000× *g* for 10 min at 4 °C, the protein content was quantified using Bradford analysis (Bio-Rad, 500-0006), and 20 μg was subjected to PAGE using an 8% denaturing polyacrylamide gel. Standard Western blotting procedures were followed to detect proteins using anti-MYC Y69 (Abcam, ab32072, 1:1000 dilution, Cambridge, UK) and anti-α-tubulin (Sigma, T9026, 1:1000 dilution, St. Louis, MO, USA) antibodies. To detect MYC levels in explanted tumors, a section of the tumor was cut and homogenized in RIPA buffer containing protease inhibitors. Preparation of protein lysates and analysis was conducted as above except blots were probed with anti-MYC N-262 antibodies (Santa Cruz, sc-764, 1:250 dilution, Santa Cruz, CA, USA).

### 4.8. Chromosome Conformation Capture

Chromosome conformation capture (3C) analysis was performed on control and 3′ WRE-Mut cell populations as previously described with minor modifications [38]. A bacterial artificial chromosome harboring the *MYC* genomic locus served as a control for the 3C-qPCR analysis. Formaldehyde cross-linked chromatin was digested with 40 μL (400 units) of BstYI (New England Biolabs) overnight at 37 °C. Primer sequences used to detect the *MYC* 5′3′ chromatin loop, and those to amplify the control region, are available in Table S1. 3C products were amplified for 32 cycles in triplicate reactions using DreamTaq polymerase (ThermoFisher, K1081). Products were resolved on a 1.5% agarose gel, imaged, and band intensities were quantified using ImageJ software. Each *MYC* 3C product was first normalized to the average value obtained from the three BAC 3C products, then to the average value of the three internal control bands.

### 4.9. Analysis of Potential Off-Targets

To screen for potential off target effects of CRISPR cleavage in 3′ WRE-Mut cells, genomic DNA was isolated from 2.5 × 10^6^ parental control HCT116 cells and 3′ WRE-Mut cells using TNES buffer. Following phenol:chloroform:isoamyl alcohol extraction and chloroform back-extraction, genomic DNA was precipitated in ethanol. Potential off-targets were identified using the CRISPR Design tool [15]. These sites were PCR-amplified using primers that flanked the target site and the products were analyzed on an 8% non-denaturing polyacrylamide gel. Primer sequences used in these reactions are listed in Table S1.

### 4.10. RNA-Seq 

RNA was extracted from two biological replicates containing 7.5 × 10^5^ control or 3′ WRE-Mut cells using the mirVana kit (ThermoFisher, AM1560). The RNAs were quantified using a NanoDrop apparatus (ThermoFisher) which also ensured that the A_260_:A_280_ ratio was 1.9 or above. The RNA integration number (RIN) for each sample was measured using an RNA 6000 Nano Kit (Agilent, 5067-1511, Santa Clara, CA, USA), and a RIN value of above 7 was required to proceed. The cDNA libraries were prepared using the NEXTflex™ Rapid RNA Sequencing Kit (BioO Scientific, 5138-02, Austin, TX, USA) and NEXTflex™ RNA-Seq Barcodes–24 Kit (BioO Scientific, 512980) following the manufacturer’s instructions. The processed libraries were assessed for fragment length size distribution and were subsequently concentrated using a BioAnalyzer High Sensitivity DNA kit (Agilent, 5067-4626). The libraries were pooled, diluted to 2 nM in elution buffer (Qiagen, 19086), and then denatured following Illumina sequencing guidelines. The denatured libraries were diluted to 10 pM in pre-chilled hybridization buffer, and loaded into a TruSeq SR v3 flow cell (TruSeq SR Cluster Kit v3-cBot™ –HS, GD-401-3001, Illumina, San Diego, CA, USA). The samples were sequenced using a HiSeq 2500 that was set to run for 50 cycles using a single-read recipe (TruSeq SBS Kit v3, FC-401-3002) according to the manufacturer’s instructions. The sequence reads that passed the default purifying setting of the Illumina CASAVA pipeline (released version 1.8, Illumina) were further trimmed/filtered by requiring a quality score cutoff of 20 using the FASTX-Toolkit [39]. The filtered reads were aligned to the human reference genome (build hg19) using Tophat v2.0.9 [40], allowing up to two mismatches. Fragments Per Kilobase Of Exon Per Million Fragments Mapped (FPKM) values were calculated using Cufflinks v2.0.2 [41] provided with the Gencode v19 gene annotation. The Gene Ontology (GO) [42] and WebGestalt [43] databases were used to classify genes that were differentially expressed in 3′ WRE-Mut *versus* control cells. The RNA-Seq data was deposited on the Gene Expression Omnibus database [44] and can be accessed using reference number, GSE70833.

### 4.11. Cell Proliferation

Approximately 7.5 × 10^4^ parental or 3′ WRE-Mut cells were seeded in duplicate wells of a 6-well plate. On days one, three, and five after plating, the cells were pelleted, stained with 0.2% trypan blue, and 20 μL of the cell suspension was loaded into a Cellometer cell-counting chamber (Nexcelom Bioscience, CHT4-SD100, Lawrence, MA, USA). Viable cells were quantified using a Cellometer Auto T4 Cell Counter (Nexcelom Bioscience).

### 4.12. Clonogenic Growth Assays

Two hundred control or 3′ WRE-Mut cells were seeded in triplicate wells of a 6-well dish and ten days later, the cells were washed twice with 1× PBS and fixed for 5 min at room temperature in 500 μL of a methanol:acetic acid solution (3:1 ratio). The cells were then stained with a 0.5% solution of crystal violet dissolved in methanol for 15 min at room temperature and washed with distilled water to remove excess stain. Following guidelines provided in Guzman *et al.* [45], stained colonies were quantified using the ColonyArea ImageJ plug-in application.

### 4.13. Anchorage-Independent Growth Assays

Anchorage-independent growth assays were performed as described previously [46] with minor modifications. Briefly, 5 × 10^3^ control or 3′ WRE-Mut cells were seeded within a 0.4% layer of agar on top of a 0.5% layer in triplicate wells of a 6-well dish. After 21 days, colonies were stained with 0.01% crystal violet and quantified using the particle analyzing function of ImageJ software.

### 4.14. Mouse Xenograft Assays

Approximately 5 × 10^6^ control or 3′ WRE-Mut cells were resuspended in 150 μL of 1X PBS and injected subcutaneously into the flanks of NU/NU nude mice (strain #088, Charles River Labs, Wilmington, MA, USA). Starting on day 10 after the injection, tumors were measured every two days using standard calipers. Volumes were determined using the formula width^2^ × length/2 [47]. At the conclusion of the experiment (day 20), the tumors were removed, weighed on a standard balance, photographed, and minced into small portions for RNA and protein analysis. The Pennsylvania State University College of Medicine Institutional Animal Care and Use Committee (IACUC) approved the animal protocols used in this study.

### 4.15. Statistical Analysis

Each experiment was repeated at least three times. For ChIP-qPCR and qRT-PCR, each sample was amplified in quadruplicate wells in each experiment. A Student’s *t*-test was used to calculate statistical significance in these experiments as well as those involving cell counts, colony growth assays, anchorage-independent growth assays, and xenograft tumor assays.

## 5. Conclusions

In this study, we have shown that the *MYC* 3’ WRE is a critical regulatory enhancer element that controls *MYC* gene expression in the HCT116 human CRC cell line. Deleting a single TBE motif within this enhancer decreased *MYC* gene expression, MYC protein levels, and the oncogenic potential of these cells. Genome-wide RNA-seq analysis found numerous differentially expressed target genes in *MYC* 3’ WRE-Mut cells compared to parental controls, with a particular enrichment for those controlling metabolic processes. These results indicate that the *MYC* 3’ WRE acts as an onco-enhancer to drive CRC.

## Supplementary Materials

The following are available online at http://www.mdpi.com/2072-6694/8/5/52/s1, Figure S1: Isolation of a second 3′ WRE-Mut clone, Figure S2: Evaluation of potential CRISPR/Cas9 off-targets, Table S1: Oligonucleotide sequences, Table S2: RNA-Seq gene lists.
