# Supplementary material for: The MYC 3′ Wnt-Responsive Element Drives Oncogenic MYC Expression in Human Colorectal Cancer Cells
Source: Cancers (Basel). 2016 May 23;8(5):52. doi: 10.3390/cancers8050052 (PMC4880869; doi:10.3390/cancers8050052)
Supplement: Supplementary file 1 [file cancers-08-00052-s001.zip › cancers-127669-supplementary-final/cancers-127669-supplementary figures-final.pdf]

# Supplementary Materials: The *MYC* 3′ Wnt-Responsive Element Drives Oncogenic *MYC* Expression in Human Colorectal Cancer Cells

**Sherri A. Rennoll, Melanie A. Eshelman, Wesley M. Raup-Konsavage, Yuka Imamura Kawasawa and Gregory S. Yochum**

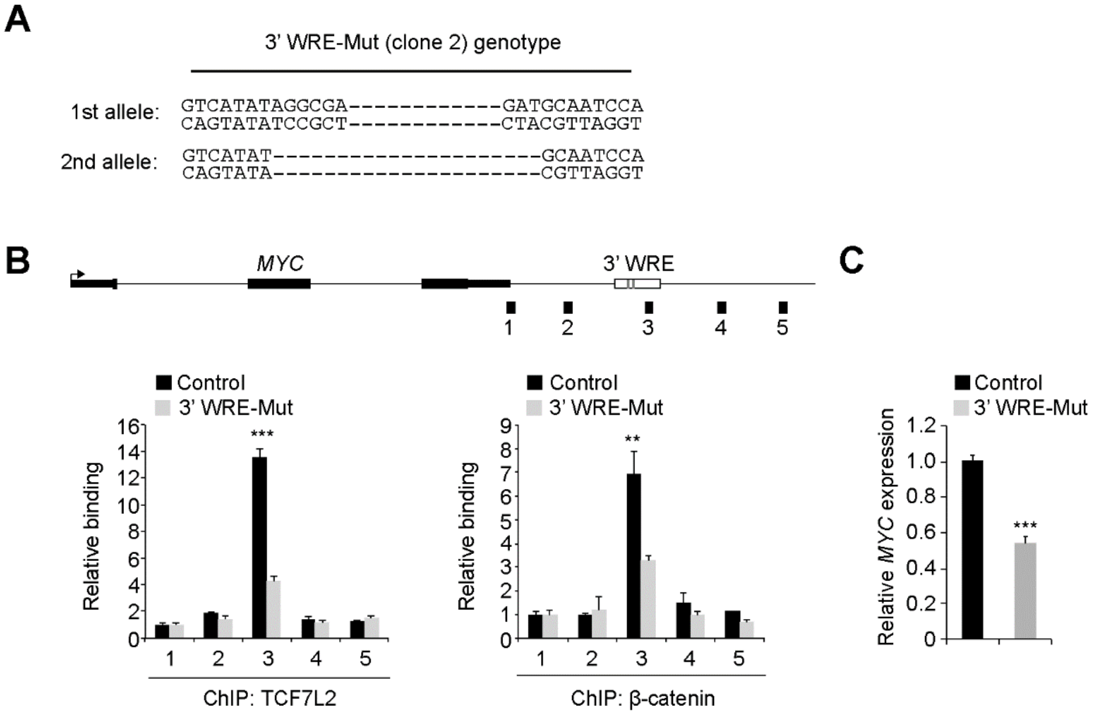


**Figure S1.** TCF7L2/β-catenin binding to the *MYC* 3′ WRE and *MYC* expression are reduced in a second 3′ WRE-Mut clonal HCT116 cell line. (**A**) Sequences of the targeted *MYC* 3′ WRE alleles in the second clone. Refer to Figure 1 for the wild-type sequence; (**B**) qPCR analysis of DNA fragments precipitated with anti-TCF7L2 or anti-β-catenin antibodies in ChIP assays conducted in control and 3′ WRE-Mut HCT116 cells. The position of the PCR amplicons used to detect binding (1-5) are indicated on the diagram of the *MYC* locus above. The signals are normalized to levels detected with amplicon 1; (**C**) qRT-PCR analysis of *MYC* transcript levels detected in control and 3′ WRE-Mut cells. The data is normalized to *GAPDH* levels. Error bars are ±SEM (** $p < 0.01$, *** $p < 0.001$).

*MYC* 3' TBE1 CRISPR target sequence: GTCATATAGGCGAATTTCAAAGG

| | | | |
|---|---|---|---|
| off-target 1 | chr8:138982738 | TACATATAGGAGAATTTCAAAGG | 3 mismatches [1, 2, 11] |
| off-target 2 | chr4:168449180 | TTCATATTGGCTAATTTCAAAAG | 3 mismatches [1, 8, 12] |
| off-target 3 | chr5: 3826037 | TTCATTTAGGAGAATTTCAATAG | 3 mismatches [1, 6, 11] |
| off-target 4 | chr10: 26930866 | TTCTTATGGGTGAATTTCAATGG | 4 mismatches [1, 4, 8, 11] |
| off-target 5 | chr9: 106293769 | TTTAGATAGGTGAATTTCAACAG | 4 mismatches [1, 3, 5, 11] |
| off-target 6 | chr21: 32202491 | GGCACATGGGTGAATTTCAAGAG | 4 mismatches [2, 5, 8, 11] |
| off-target 7 | chr12: 77224237 | GCCATATGGAAGAATTTCAATAG | 4 mismatches [2, 8, 10, 11] |
| off-target 8 | chr1: 246908861 | GGGATATAGCTGAATTTCAAGGG | 4 mismatches [2, 3, 10, 11] |
| off-target 9 | chr7: 128780931 | GAAATATGGGCCAATTTCAACAG | 4 mismatches [2, 3, 8, 12] |
| off-target 10 | chr5: 85292377 | ATTATAGAGGAGAATTTCAAAAG | 4 mismatches [1, 3, 7, 11] |
| FAM112A | chr20: 42355297 | ATCATAAGGGCAAATTTCAAAGG | 4 mismatches [1, 7, 8, 12] |
| GNA13 | chr17: 63007955 | TTCATATAGTGGTATTTCAAAAG | 4 mismatches [1, 10, 11, 13] |
| KCNH7 | chr2: 163256716 | GTCATCTTTGAGAATTTCAATGG | 4 mismatches [6, 8, 9, 11] |
| MYO1E | chr15: 59664763 | GTCTTCTGGGCGAACTTCAAAGG | 4 mismatches [4, 6, 8, 15] |
| ALDH1L1 | chr3: 125850152 | GCCATATAGGAGAACTCCAAGGG | 4 mismatches [2, 11, 15, 17] |
| CSMD3 | chr8: 113237073 | GTCATACATGGGATTTTCAAAAG | 4 mismatches [7, 9, 11, 14] |

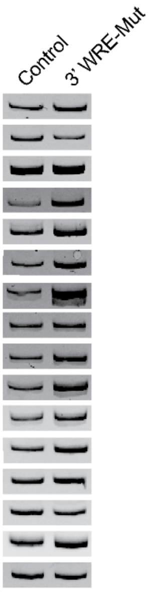


**Figure S2.** Insertions or deletions (indels) are absent at the top predicted off-targets in 3′ WRE-Mut cells. PAGE analysis of PCR products amplified from genomic DNA isolated from parental HCT116 (control) or 3′ WRE-Mut cells using primers that anneal to regions flanking the putative off-target sites. Listed are the targets analyzed and their chromosomal position. Nucleotides that are not conserved in the *MYC* 3′ WRE CRISPR target sequence are in red. The PAM sequence is underlined.
